# Rev. Legh Richmond's Statement Respecting Ann Moore

**Published:** 1813-06

**Authors:** Legh Richmond

**Affiliations:** Rector of Turvey, Bedfordshire


### *To the Editors of the Medical and Physical Journal.*

GENTLEMEN,

ALTHOUGH I am a member of a different profession from that of the correspondents whose valuable researches and labors *usually* enrich your publication, yet I trust you will not, on the present occasion, think me intrusive in sending you a plain statement of facts connected with the detection of the imposture of Ann Moore, of Tutbury. The subject has been commented upon in your pages at different periods by gentlemen of medical and physiological attainments, who have considered the case in very opposite points of view, as to the possibility and probability of human life being capable of support without nourishment by the ordinary channels and organs of digestion. It seems, therefore, desirable that the result of an actual and decisive investigation, originating with and instituted by myself, and since carried into execution by the assistance of a committee of gentlemen of the first respectability, should now be made public through the same medium of communication. Science always gains by the establishment of facts. Hypotheses, however ingenious; surmises, however plausible; of necessity leave subjects, of a disputable and disputed nature, still in a state of doubt and uncertainty. But when we attain to the possession of demonstrative evidence, positive acquirements are made to the cause of truth and knowledge, both moral and physical.

The case of Ann Moore had attracted the attention not only of the vulgar and more ignorant part of the community, but of very many intelligent and thinking persons. This arose not so much from the extraordinary nature of the woman's own assertion, or from any puerile inclination to yield implicit credit to what such a person, from interested motives, might think proper to declare, as from an importance attached to the case by the sanction which the supposed possibility and and even probability of her story being valid, had obtained from numerous medical, philosophical, and well-informed characters, who, after visiting her, and investigating the circumstantial evidence of the history, admitted that there were strong grounds for giving assent to the truth of her declarations.

Some have, at different periods, altered their opinions, and that on each side of the question. Others have uniformly expressed doubts of the reality of *her* case in particular, but by no means denied the possibility of its existence. Some have formed and published hypotheses as to the mode of her supposed subsistence. The question as to the *mode* has been debated in different philosophical societies. Various parallel cases of long abstinence, yet uncontradicted or undetected, have been made public and referred to in illustration of that at Tutbury. It was, therefore, apprehended by many, and those of no little estimation in the scale of talent, science, and general respectability, that, as the *first* watch, instituted on this woman four years and a half since, was supposed to be decisive in her favor, and as no testimony of a conclusive nature was afterwards ever known to be brought against her, there was something sufficiently interesting in her case to justify, at least, a conditional assent to its truth, and, at all events, a minuter examination of its evidences.

I first went, as a stranger from a distant county, to visit her in the month of August 1810, remained about an hour in her apartment, heard her own account, compared it with various medical and other attestations of facts, made some inquiries amongst the inhabitants of the place, and came away under an impression of hope that her *apparently* solemn declarations were not false; the more so as there appeared to be evidences, both scientific and circumstantial, in her favor.

During the two following years, it often occurred to me that her story ought to be more completely investigated, either for the undeniable establishment of the singular fact, if true; or the full detection of the infamous deception, if false. It appeared to me desirable that the matter should be publicly authenticated or disproved; and, as no one had yet, in any decisive manner, undertaken the examination of the case and its validity, I took the opportunity of a journey through a neighbouring county, to visit Tutbury on the 16th of last November. My object was to inquire more accurately into all the circumstances, and to propose her undergoing a *second* watch, to be conducted, if possible, on a conclusive principle. I was accompanied by a clerical friend, and a gentleman from London. I shall not enter into into any detail of that imposing cast of conversation which she, with no small art and ability, had always at command. It may suffice to say, that she heard my proposition with seeming complacency, admitted the propriety of the test, stated her objection to the *manner* in which the same thing had been proposed to her by others, and finally gave her full consent to undergo a second watch under my conduct and superintendence. She then expressed her particular desire that the persons selected to be her watchers should be clergymen and medical men only, if such could be obtained. She stipulated that she should be treated during the watch with humanity and tenderness, but without the slightest abatement from the most scrupulous vigilance of scrutiny as to the fact whether she took any food or not. The period *then* appointed for the watch to continue, was three weeks. The gentlemen present were witnesses to her promise of submitting to the trial of this watch, if I would undertake the execution of it. I stated that early in the spring I hoped to be at liberty to proceed in the business, and would take the necessary steps for its accomplishment.

In the month of January, I was informed by a gentleman of Tutbury that she persevered with alacrity in the prospect of the proposed experiment. I had corresponded with different friends on the preparatory measures, and, finding that the project had attracted extensive notice and approbation, I attended a meeting of magistrates, clergymen, medical and other gentlemen, who assembled at Tutbury on March 20th last, in order to carry into effect the watch which I had, in the November preceding, undertaken, if possible, to accomplish. A committee was formed, consisting of gentlemen of the first respectability, in order that the watch might be superintended in the most satisfactory manner. Various regulations were formed. It was resolved that watchers should be invited from the three classes of magistrates, clergymen, and medical gentlemen. This was done by public advertisement. Numerous offers of assistance were speedily made from gentlemen of the first estimation, far and near. The proposed period of the watch was lengthened to a month. Impartiality and activity were exhibited in every part of the transaction. Every thing which could tend to satisfy the minds of the most accurate observers, was pursued at the commencement of the watch. Her bedstead, made for the occasion under the management of the committee, was placed on a Merlin's weighing machine, in order to ascertain the progressive variations of her weight. Complete examination of the room, walls, floor, and furniture, took place in the presence of the committee and the first

1 watchers.

watchers. A new bed was filled in their presence, the bedding was examined, and her removal from one to the other accurately superintended. Her person and dress were submitted to inspection, and nothing left in any state of doubt and uncertainty.

The watch commenced on Wednesday, April 21, at two o'clock in the afternoon, and was continued with strict and unabated vigor till Friday the 30th, at two o'clock in the morning. A cold, which she caught during the removal from her own bed on the first day of the watch, produced for several days symptoms of catarrh, fever, expectoration, &c. which in some measure interfered with so accurate an observation on the effects ascribable to abstinence only, as was desirable. Many remarks and notes were, however, made by the committee, and different watchers, which, with various particulars of the transaction, will be communicated to the public in a pamphlet preparing for the press. On the latter days of the watch, symptoms of a description not to be accounted for by catarrhal fever alone, were discovered, although a difference of opinion existed amongst the medical gentlemen as to their actual cause. Great emaciation and loss of weight took place. On Friday morning, April 30th, her livid, weak, and wasted appearance, the state of her pulse, and other concomitant appearances, induced the physicians and surgeons who were called in, to pronounce her in great danger of dying speedily. She desired that the watch might for the present cease, and stated a readiness to be watched again if she recovered from her present illness. The opinion of the medical attendants under these circumstances, led the committee to think it right to comply with her request, and the watch accordingly ceased at twelve at noon. But, in fact, during the few hours preceding its formal conclusion, two medical gentlemen had suggested the necessity and propriety of giving her cloths dipped in vinegar and water, to wash her tongue and fauces from the mucus with which she was oppressed, and to cool a raging heat. As a case of apparent necessity, they accordingly administered it to her; and, in fact, one of them by this means thought he discovered an attempt at deglutition. Of course the virtual period of the conclusion of the watch must be dated from the moment of this communication of the vinegar and water.

Although apparently dying, in the estimation of the faculty, previous to the breaking up of the watch, she revived afterwards in a few hours, having evidently received strength through the communication of the liquid, although she still denied having swallowed any of it.

But

But subsequent evacuations of urine, and the discovery made by some gentlemen of the committee as to the state of her linen, clearly proved that she had not only swallowed liquid immediately before or after the conclusion of the watch, when her family had free access to her, but that she must have also taken *liquid* at least previous to the original commencement of the watch. During these discoveries she equivocated and contradicted herself: at length, being confronted with evidence which she could not deny, she confessed before a magistrate, who has attested her confession, that, contrary to her well-known assertion, so long persevered in, she *had* occasionally taken sustenance during the last six years.

This is not the time to dwell upon the moral depravity which the circumstances of this detection have brought to light. Remarks of this nature shall be reserved for another occasion: they do, however, constitute no small part of the feelings and observations of many witnesses to all the steps of the transaction.

She still persists in asserting that she possesses very extraordinary powers of abstinence. Of the truth of this, from the circumstances of the late watch, and other previous events, there is considerable evidence. But, after so full a detection of her deliberate falsehood on the point of *total* abstinence, no credit can attach to any declarations, except such as may be substantiated by more credible witnesses than herself, or those immediately connected with her.

I refrain from recording such medical particulars as will more properly and authoritatively come from the pens of gentlemen of the medical profession, who will, probably, communicate their observations to the public. My simple object has been to state a few of the leading facts which tended to unveil the case of an impostor, who has been no less remarkable for the boldness of her assertions, than for the success with which they have been attended.

Whatever may be the opinions of gentlemen distinguished for acuteness of research and respectability of attainments, who have stated their sentiments as to the *possibility* of human subsistence under circumstances of extreme or total abstinence from food; the present detection sets the question at rest so far as the case of Ann Moore is concerned. What degree of assent may be due to several other instances of abstinence recorded in various literary, medical, and philosophical journals, I pretend not to decide. A scientific investigation into all the *apparently* well-authenticated cases of a similar nature, with physiological remarks on the nature of abstinence, and its acknowledged effects, may be worthy of the consideration of some of the able and intelligent writers of the present age.

It is but an act of justice to the peculiarly active and impartial conduct of those members of the committee who are inhabitants of Tutbury, to state, that to *their* individual anxiety and exertion to prove or disprove the truth of a case which has, in some instances, been very illiberally and unjustifiably suffered to implicate the character of the inhabitants of the place in general, the circumstances of final detection are in a particular degree owing. The same remark is justly due to the feelings, sentiments, and behaviour, manifested by the people of the place and neighbourhood at large, since the discovery was made.

Once more requesting your indulgence for this introduction of myself into your publication, allow me to say that I am thankful to have been in any measure instrumental to the train of events which have led, in the present instance, to the discovery of truth and the detection of falsehood, and am

Your obedient Servant,

LEGH RICHMOND,

*May* 10, 1813. Rector of Turvey, Bedfordshire.

